# Comparison of Brazilian Plants Used to Treat Gastritis on the Oxidative Burst of *Helicobacter pylori*-Stimulated Neutrophil

**DOI:** 10.1155/2013/851621

**Published:** 2013-07-18

**Authors:** Cibele Bonacorsi, Luiz Marcos da Fonseca, Maria Stella Gonçalves Raddi, Rodrigo Rezende Kitagawa, Wagner Vilegas

**Affiliations:** ^1^Instituto de Ciências da Saúde, Universidade Federal de Mato Grosso, 78557-267 Sinop, MT, Brazil; ^2^Faculdade de Ciências Farmacêuticas, Universidade Estadual Paulista (UNESP), 14801-902 Araraquara, SP, Brazil; ^3^Departamento de Ciências Farmacêuticas, Universidade Federal do Espírito Santo, 29040-090 Vitória, ES, Brazil; ^4^Campus Experimental do Litoral Paulista, Universidade Estadual Paulista (UNESP), 11330-900 São Vicente, SP, Brazil

## Abstract

Ten Brazilian medicinal plants used to treat gastritis and ulcers were carefully selected on the basis of ethnopharmacological importance and antiulcerogenic activity previously described. The antioxidant activity of the methanolic extracts was determined in analysis conditions that simulate a real biological activity on inhibition of the oxidative burst induced in neutrophils using *Helicobacter pylori* as activator, by a luminol-amplified chemiluminescence assay. The extracts, at low concentration (5 **μ**g/mL), exhibited a large variation in inhibitory effects of *H. pylori*-induced oxidative burst ranging from 48% inhibition to inactive, but all extracts, excluding *Byrsonima intermedia*, had inhibitory activity over 80% at the concentration of 100 **μ**g/mL. The total suppressive antioxidant capacity measured as the effective concentration, which represents the extract concentration producing 50% inhibition of the chemiluminescence induced by *H. pylori*, varies from 27.2 to 56.8 **μ**g/mL and was in the following order: *Qualea parviflora > Qualea multiflora > Alchornea triplinervia > Qualea grandiflora > Anacardium humile > Davilla elliptica > Mouriri pusa > Byrsonima basiloba > Alchornea glandulosa > Byrsonima intermedia*. The main groups of compounds in tested extracts are presented. Differences in the phytochemical profile, quantitatively and qualitatively, of these plants can explain and justify their protective effect on the gastric mucosa caused by the neutrophil-generated ROS that occurs when *H. pylori* displays its evasion mechanisms.

## 1. Introduction

*Helicobacter pylori* infection is recognized as an important causative agent of gastroduodenal diseases, causing chronic gastritis, peptic ulcer disease, and increased risk of cancer [1]. *H. pylori* infects about one-half of the world's population and usually persists lifelong unless eradicated by antibiotic treatment [2]. In Brazil, the overall prevalence of *H. pylori* is high when compared to that of developed countries [3]. The rate of *H. pylori* prevalence in adults in the south of Brazil is about 63% [4], while in a poor urban community in northeastern it is 80% [5]. 

Infiltration of the gastric mucosa with neutrophils, macrophages, and lymphocytes is a hallmark of *H. pylori* infection [6]. To generate the microbicidal oxidants, polymorphonuclear neutrophils demonstrate a burst of oxidative activity, which is known as the respiratory burst, by releasing large quantities of superoxide anion (O_2_^−^) as a result of the activation of NADPH oxidase [7]. The enzyme superoxide dismutase reduces the superoxide anion radical to form hydrogen peroxide (H_2_O_2_) and oxygen (O_2_) [8]. Myeloperoxidase, an enzyme released from the azurophilic granules in neutrophils, uses H_2_O_2_ and chloride ions (Cl^−^) as substrates to produce hypochlorous acid (HOCl), an important antibacterial compound, but an extremely strong oxidant that can also attack host biomolecules [9]. Although the neutrophils recruited to the gastric mucosa during infection represent one obvious source of oxidative stress, *H. pylori* itself also generates reactive oxygen species (ROS) to resist oxidative damage from chronic inflammation that accumulates in gastric epithelial cells [10]. Paradoxically, this robust immune/inflammatory response cannot clear the infection, thus leaves the host prone to complications resulting from chronic inflammation. In addition, extensive studies have revealed that *H. pylori*-induced ROS production in gastric epithelial cells might affect gastric epithelial cell signal transduction, resulting in gastric carcinogenesis [10]. 

In Brazil, a large number of herbal extracts are used in folk medicine to treat various types of digestive disorders, and several studies have documented the beneficial effects of Brazilian plant in the prevention of gastric injury [11–19]. The mechanisms by which *H. pylori* infection leads to gastric mucosal damage include the direct effects of virulent factors produced by bacterium, the propagation and perpetuation of inflammation, oxidative stress, and the induction of apoptosis in infected gastric epithelial cells [20]. Herbs that can protect cells from oxidative stress and antioxidant therapeutic approaches may play an important role by protecting the gastric mucosa from oxidative damage or by accelerating healing of gastric ulcers [21]. 

In our previous study, the antioxidant activity of some Brazilian plants was investigated in a cell-free system using the DPPH radical scavenging activity [22]. The DPPH assay is classified as a single-electron transfer reaction that may be neutralized by the antioxidant either by direct reduction via electron transfers or by radical quenching via H atom transfer [23]. One of the limitations of this assay is the poor correlation between DPPH chemical structure with free radicals produced in biological systems [24]. Therefore, to study the potential antioxidant effects of natural products, it is very important to add a cellular-based assay considering the complexity involved in their *in vivo* mechanisms of action [25].

Chemiluminescence assays that measure the production of ROS have been widely used as a sensitive assay for monitoring free radicals and reactive metabolites produced by enzymes, cells, or organ systems [26]. The use of chemiluminescent probes amplifies chemiluminescence by allowing the detection of low levels of light emission, thereby increases the sensitivity of the reaction [27]. Antioxidants affect the intensity of luminol-dependent chemiluminescence [28]. Since the release of ROS by polymorphonuclear, neutrophils are believed to be an important part of the pathogenesis of *H. pylori*-associated gastritis and duodenal ulcer are; in the present study, the methanolic extracts (MeOH) obtained from Brazilian medicinal plants used to treat gastritis and ulcer were investigated for their antioxidants effects on the neutrophil oxidative burst generated by *H. pylori* as a stimulant, using luminol-amplified chemiluminescence. This study is a part of a larger survey in which other functional properties of these extracts such as their antimicrobial, antiinflammatory, and antiulcerogenic activities were also evaluated [11–19].

## 2. Material and Methods

### 2.1. Plant Material and Preparation of the Extracts

 The MeOH extracts used in this study from *Alchornea glandulosa* (Euphorbiaceae) [11], *Alchornea triplinervia* (Euphorbiaceae) [12], *Anacardium humile* (Anacardiaceae) [13], *Byrsonima basiloba*, *Byrsonima intermedia* (Malpighiaceae) [14], *Davilla elliptica* (Dilleniaceae) [15], *Qualea grandiflora* (Vochysiaceae) [16], *Qualea parviflora* (Vochysiaceae) [17], *Qualea multiflora* (Vochysiaceae), and *Mouriri pusa* (Melastomataceae) [18, 19] were the same as the used in our previous studies. Briefly, the dried powdered plant material (leaves or bark) was extracted exhaustively with successions of methanol at room temperature. The extract was filtered and concentrated under reduced pressure at 60°C with a rotary evaporator to yield the MeOH extract. The reference material, extract, and phytochemical screening of these plants are described in Bonacorsi et al. [22].

### 2.2. Animals

Male rats (*Rattus norvegicus albinus*) weighing 290 ± 20 g were obtained from the Animal House of the São Paulo State University “Júlio de Mesquita Filho” (UNESP). The animals were maintained at 23 ± 2°C and a relative humidity of 50 ± 5% under a 12 h light/12 h dark cycle. The Ethical Committee of the Pharmaceutical Sciences at Araraquara—UNESP—approved the experimental procedure of this study (resol 05/2008).

### 2.3. Experimental Protocols

#### 2.3.1. Collection of Polymorphonuclear Neutrophils

Suspensions of polymorphonuclear neutrophils were obtained from rats by intraperitoneal (i.p.) injection of 10 mL of a solution of sterile oyster glycogen 0.5% (w/v) in saline followed, 12 h later, by lavage with 20 mL Dulbecco's phosphate-buffered saline (D-PBS) without calcium containing 10 IU heparin/mL. The cells were washed twice in D-PBS and were carefully layered onto 5 mL of Ficoll-Paque (*d* = 1077) and centrifuged at 800 ×g for 30 min. Subsequently, the neutrophils were washed again with D-PBS and adjusted to a concentration of 2.0 × 10^6^ cells/mL. The proportion of neutrophils (over 95%) and cell viability in the peritoneal exudate were determined by cell staining with May-Grünwald-Giemsa.

#### 2.3.2. Helicobacter Pylori Strain, Culture Conditions, and Luminol Chemiluminescence Assay

*H. pylori* type strain ATCC 43504, which is metronidazole resistant and amoxicillin susceptible, was obtained from the American Type Culture Collection (Manassas, VA, USA). The bacterium was grown in a microaerophilic atmosphere at 37°C on Columbia agar containing 5% sheep blood for 3 days. *H. pylori* organisms were collected and suspended in 0.01 M phosphate buffered 0.15 M saline, pH 7.4 (PBS) at different absorbance (0.15, 0.2, and 0.3) with a 620 nm filter in order to follow the respiratory burst of polymorphonuclear neutrophils exposed to bacteria using luminol-enhanced chemiluminescence assay performed following the protocol described by Bonacorsi et al. [29]. Briefly, 5.0 × 10^6^ cells/mL and 2.0 × 10^−5^ M luminol (Sigma Chemical Co., St. Louis, MO) were added to a tube containing PBS-D. This vial was placed in a lightproof chamber of a Bio-Orbit model 1251 luminometer (Bio-Orbit, Finland), and the carousel was rotated to bring the sample in line with the photomultiplier tube to record background activity. The control stimulus (1 mg/mL zymozan A (Sigma Chemical Co., St. Louis, MO)) or bacteria were added to the suspension at a final volume of 1.0 mL. The chemiluminescence emission of each vial, expressed in mV, was recorded for 90 min to obtain kinetic curves. All experiments were carried out in triplicate and repeated at least three times. 

#### 2.3.3. Luminol-Dependent Chemiluminescence for Determination of Total Antioxidant Reactivity of Extracts

The inhibitory effect of the MEOH extracts on the chemiluminescence emission by *H. pylori*-stimulated neutrophils was determined using the chemiluminescence assay described above. For the inhibition experiments, the stimulus (*H. pylori* suspension at an optical density of 0.2) was added to the tubes, and light release (in mV) was measured for 15 min. After this, D-PBS containing the MEOH extract at noncytotoxic concentrations (5, 50, and 100 *μ*g/mL) was added, and the oxidative burst was continuously monitored for another 75 min. The chemiluminescence response was quantified as the integrated area below the resulting chemiluminescence curve, over a period from 0 to 90 min. The background chemiluminescence from neutrophils in the absence of stimulus was also measured. The effective concentration (EC_50_), that is, the extract concentration producing 50% inhibition of the chemiluminescence induced by *H. pylori*, was calculated using a log-plot transformation of the data. Quercetin was used as a standard antioxidant. All tests were performed in triplicate and repeated at least three times. None of these extracts affected neutrophils viability (over 95%) upon 90 min incubation with 100 *μ*g/mL of plant extracts.

### 2.4. Statistical Analysis

The parameters were expressed as the mean (SD). Data were analyzed by analysis of variance (ANOVA). The differences were considered statistically significant when the test yielded a value of *P* ≤ 0.05 compared to the standard antioxidant.

## 3. Results

**Figure 1:**
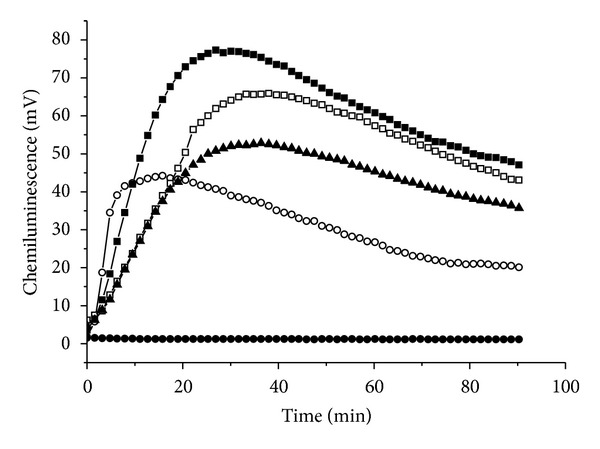
Luminol-enhanced chemiluminescence kinetic profile of stimulated neutrophils with *Helicobacter pylori* and nonopsonized zymosan. Background (solid circles), zymosan 700 *μ*g/mL (open circles), *H. pylori* suspension 0.15 ODU (solid triangles), *H. pylori* suspension 0.20 ODU (solid rectangles (■)), and *H. pylori* suspension 0.30 ODU (open rectangles). Each curve was constructed from 90 points, each point representing the mean of three replicates.

Challenge of neuthrophils with *H. pylori* resulted in a strong luminol chemiluminescence response (exemplified in Figure 1). It was found that a significant kinetic profile occurs following exposures of neutrophils to different concentrations of *H. pylori*.* H. pylori* suspension 0.20 ODU was used in the tests. 

The capacity of the MEOH extracts to inhibit the oxidative burst induced by *H. pylori* is shown in Table 1. The extracts inhibited the oxidative burst in a concentration-dependent manner, that is, as the concentration of the sample increased, the percentage inhibition of oxidative burst also increased to a certain extent and then leveled off with further increase in extracts concentration.

The results revealed that the tested extracts, at low concentration, have a large variation in antioxidant activity ranging from 48% to inactive at 5 *μ*g/mL. The inhibition of the oxidative burst of these plant extracts was in the following order: *Qualea parviflora* > *Qualea multiflora* > *Alchornea triplinervia* > *Alchornea glandulosa *> *Byrsonima intermedia * > *Qualea grandiflora *> *Anacardium humile * > *Davilla elliptica*; *Mouriri pusa* and *Byrsonima basiloba* were inactive. All examined extracts, excluding *B. intermedia*, had antioxidant activity over 80% at the concentration of 100 *μ*g/mL. In terms of efficient concentration, the EC_50_ values ranged from 27.2 to 56.8 *μ*g/mL. The suppressive activity was in the following order: *Qualea parviflora* > *Qualea multiflora* > *Alchornea triplinervia* > *Qualea grandiflora* > *Anacardium humile* > *Davilla elliptica* > *Mouriri pusa* > *Byrsonima basiloba* > *Alchornea glandulosa* > *Byrsonima intermedia*. Although the EC_50_ for *A. glandulosa *and *A. humile* is close (43.4 and 40.2 *μ*g/mL, resp.), *A. humile *was less active than *A. glandulosa* at 5 *μ*g/mL (9.1% and 21.6%, resp.). *B. basiloba* and *M. pusa* were inactive at the 5 *μ*g/mL but they showed high activity at the concentration of 100 *μ*g/mL (93.4% and 93.0% inhibition). 

## 4. Discussion

Despite years of experience with *Helicobacter pylori* treatment, the ideal regimen for treating this infection remains to be found [1]. Antibiotics are not the only factor of the success of *H. pylori* eradication therapy; another factor influencing *H. pylori* eradication rate is microenvironment created by the bacteria. The more severe clinical manifestation associated with some *H. pylori* strains may be attributed to the higher grade of inflammation that they induce [10]. It has been suggested that antioxidants increase the effectiveness of the antibiotics by reducing inflammation and oxidative stress in the gastric mucosa [30]. Determination of the antioxidant activity of plant extracts and compounds often gives different results since the methods used are based on different reaction mechanisms [31].

The main aim of this paper was to study in analysis conditions that simulate, as much as possible, a real antioxidant activity of some Brazilian medicinal species on ROS induced in neutrophils exposure to *H. pylori* in order to give a contribution to the pharmacological validation for their use to treat ulcers and gastritis. The antioxidant capacity of MeOH extracts on the neutrophil oxidative burst was evaluated through chemiluminescence assay using luminol as probe. The chemiluminescence method is a direct method of radical investigation, though the advantage of the method consists in the fact that chemiluminescence intensity is directly proportional to a steady-state concentration of the radicals responsible for luminescence irrespective of the activity of these radicals [32]. It is enable to measure the level of free radicals and estimate antioxidant protection parameters and antioxidant action. Luminol tracks the production of reactive oxygen species formed in the intra- and extracellular environment, such as HOCl, H_2_O_2_, and O_2_^−^ [33]. From the results showed in Table 1, the extracts inhibited the respiratory burst of neutrophils induced by *H. pylori* in concentration-dependent manner.

In nature, there are a wide variety of natural antioxidants which are different in their composition, physical and chemical properties, mechanism, and site of action [34]. Among the extracts, *Qualea parviflora *and* Qualea multiflora* were found to be the most potent as they showed inhibition ranges between 48% and 95% for the tested concentrations (5 and 100 *μ*g/mL, resp.). The action mechanisms involved in the gastroprotective effects from *Qualea parviflora* consist in reducing the gastric lesion by increasing the antioxidant capacity of the gastric mucosa, though, in turn, maintaining the GSH levels, increasing sulfhydryl compounds, and stimulating the gastric PGE2 synthesis [17]. *Qualea parviflora* has been found to contain ellagic acid as its major constituent. Phytochemical studies have shown that methanolic extract contains 3,3′-di-O-methylellagic acid-4-O-beta-D-glucopyranoside, 3-O-methylellagic acid-4′-O-alpha-L-rhamnopyranoside, 3,3-4-tri-O-methylellagic acid-4′-O-beta-D-glucopyranoside, 3,3′-di-O-methylellagic acid, triterpenes, and saponins [35]. Gastroprotective properties of ellagic acids were evaluated by Beserra et al. [36] for gastric ulceration caused by ethanol, indomethacin, and acetic acid treatments. 

*Qualea grandiflora* showed a strong antiulcer effect on the surface of the gastric mucosa and the phytochemical investigation proved the presence of tannins, catechins, steroids, terpenoids, and saponins [16]. Tannins and flavanoids contain a variety of phenolic hydroxyl groups and show the strongest antioxidant capacity and free radical-scavenging activity among around a hundred phenolic compounds [37, 38]. The basic structural orientation of the compounds determines the antioxidant activity of phenolics, such as how easily a hydrogen atom from a hydroxyl group can be donated to a free radical, and the ability of the compounds to support an unpaired electron [39]. The position of hydroxyl groups seems more important than their number for the antioxidant capacity of phenolics; for example, hydroxyl groups in the ortho position of the B ring can greatly enhance the antioxidant capacity, such as in catechins [40].

The leaves of *Alchornea triplinervia *are commonly used in Brazilian folk medicine in tea form to treat gastric disturbances. In order to better comprehend the effect of MEOH extract on gastric injuries, Lima et al. [12] separated the MEOH extract into ethyl acetate and water, thus obtaining two fractions. Oral pretreatment rats with ethyl acetate fraction decreased the gastric injuries induced by ethanol resulting in more efficient gastroprotective effect than with MEOH extract. The authors observed that the ethyl acetate fraction contains primarily five phenolic compounds: ellagic acid, quercetin-3-O-beta-D-galactopyranoside, quercetin-7-O-beta-D-glucopyranoside, quercetin-3-O-beta-D-glucopyranoside, and quercetin-3-O-alpha-L-arabinopyranoside.

Higher level of gallic acid derivatives than catechins and flavonoids was detected in *Anacardium humile*, and methyl gallate has been found to be the major component [13]. Gallic acid and its derivatives are commonly used as food additives as antioxidants [41]. Plants containing substances like quercetin and gallic acid are effective in preventing ulcers, mainly because of their antioxidant properties [42]. 

According to ethnopharmacological studies, infusion from leaves of *Davilla elliptica* is employed in folk medicine as tea form to treat gastric pain, diarrhea, inflammation, and ulcer. The chromatographic profile obtained by HPLC-PAD analyses led to the recognition of three main classes of secondary metabolites in the methanolic extracts from leaves of *Davilla elliptica*: phenolic acid derivatives, flavonoids, and condensed tannins, myricetin-3-O-alpha-rhamnopyranoside has been reported to be the main flavonoid with percentage of 36.9% of the total flavonoid content; however, the most abundant class of secondary metabolites found was the condensed tannins (41.2%) [15]. Tannins are potent scavengers of peroxyl radicals, and they can also interact with mucus proteins, improving their cytoprotective effect by forming a protein lining over the gastrointestinal mucosa [43].

*Mouriri pusa* was effective in experimentally healing rat ulcers after 14 or 30 days of treatment [19]. Phytochemical investigation of the MeOH extract of *Mouriri pusa* yielded tannins, flavonoids, and (−)-epicatechin. The effect of tannins and flavonoids fractions from *Mouriri pusa* leaves methanolic extract on the prevention and cicatrisation process of gastric ulcers was also demonstrated [18]. 

*Byrsonima basiloba* is a native arboreal type from the Brazilian “cerrado” (tropical American savanna), and the local population uses it to treat diseases, such as diarrhea and gastric ulcer. Phytochemical analysis of the extracts revealed the presence of n-alkanes, lupeol, ursolic and oleanolic acid, (+)-catechin, quercetin-3-O-alpha-L-arabinopyranoside, gallic acid, methyl gallate, amentoflavone, quercetin, quercetin-3-O-(2′′-O-galloyl)-beta-D-galactopyranoside, and quercetin-3-O-(2′′-O-galloyl)-alpha-L-arabinopyranoside [44]. 

Previous investigations regarding the chemical composition of *Byrsonima intermedia* leaves indicated the presence of quercetin-3-O-beta-D-galactopyranoside, (+)-catechin, (−)-epicatechin, gallic acid, methyl gallate, quercetin-3-O-alpha-L-arabinopyranoside, and amentoflavone [45]. Since most medicinal herbs are prepared for consumption of herbal tea, Rinaldo et al. [46] have evaluated the difference between the methanolic extract and the infusible form from *Byrsonima intermedia* and showed that the extract presents higher amounts of flavan-3-ols than the infusible form per gram of leaves. Gupta and Sharma [34] provided evidence that the hot water (80°C) extraction is a useful method with extracting efficiency of 83.7% for antioxidant activity and of 77.4% for total phenolic content, as compared with 80% methanolic extraction.

Although, the phytochemical investigation of *A. glandulosa* led to the isolation of phenolic compounds like quercetin, gallic acid, amentoflavone, methyl gallate, myricetin-3-O-alpha-L-rhamnopyranoside, quercetin-3-O-alpha-L-arabinopyranoside, quercetin-3-O-beta-D-galactopyranoside, and pterogynidine [11], this extract reached an EC_50_ of 43.4 *μ*g/mL higher than that observed for *A. triplinervia*. The differences in the chemical constituents as well as in the quantity of several components might justify differences in the antioxidant activity. Bonacorsi et al. [29], under the same working conditions used in this study, reported the inhibition of luminol oxidation on neutrophil oxidative burst generated by *H. pylori* by some phenolic compounds. At 5 *μ*g/mL concentration, the most potent inhibitor was methyl gallate (73.1%) compared to (+)-catechin (28.3%), amentoflavone (16.7%), quercetin 3-O-alpha-L-arabinopyranoside (8.9%), and quercetin 3-O-beta-D-galactopyranoside (7.2%). Although the antioxidant activity of constituents of plant extracts can be demonstrated, the possible synergistic effects of these compounds should be considered.

Even though intensive studies on the chemical contents in numerous Brazilian plants commonly used in folk medicine to the treatment of gastritis and ulcers have been conducted, the complete composition data are yet insufficient to predict the antioxidant activity on the oxidative burst induced by *H. pylori *in neutrophils. There is enough evidence to conclude that these Brazilian plants might exert a beneficial effect in gastric diseases related to generation of reactive oxygen species. The use of medicinal plants as phytoceuticals or in combination with antibiotics for the treatment of *H. pylori* is an active research field.

## 5. Conclusions

As part of a continuous study on the benefits of medicinal plants regarding the gastrointestinal tract, in this work, we demonstrated the protective effect of some Brazilian plants by which *H. pylori* and neutrophils collaborate to cause gastric mucosal damage. Although all studied plants showed antioxidant activity, there are enough evidences to conclude that the most effective species confirmed for their lowest antioxidant efficient concentration were *Qualea parviflora *and *Qualea multiflora*. The present results justify the ethnomedical use of these plants, which appears to have a great potential to become useful as phytodrugs for the treatment of gastric ulcers induced by *H. pylori*.

## Figures and Tables

**Table 1:** Effects of methanolic extract of Brazilian plants used to treat gastritis on luminol-dependent chemiluminescence response of neutrophil stimulated by *H. pylori*.

Botanical name (popular name)	Concentration (*µ*g/mL)	IA^a^	% reduction in IA^b^	EC_50_^c^ (*µ*g/mL)
*Alchornea glandulosa * (tapiá)	0 (control)	2.96 × 10^5^ ± 12542	—	43.4
	5	2.32 × 10^5^ ± 9838	21.6*	
	50	0.54 × 10^5^ ± 2279	81.6*	
	100	0.52 × 10^5^ ± 2197	82.4*	

*Alchornea triplinervia* (tanheiro)	0 (control)	2.34 × 10^5^ ± 11565	—	36.6
	5	1.67 × 10^5^ ± 8245	28.6*	
	50	0.26 × 10^5^ ± 1301	88.9*	
	100	0.23 × 10^5^ ± 1144	90.2*	

*Anacardium humile* (cajuzinho-do-cerrado)	0 (control)	2.20 × 10^5^ ± 13718	—	40.2
	5	2.00 × 10^5^ ± 8077	9.1	
	50	0.21 × 10^5^ ± 2047	90.5*	
	100	0.11 × 10^5^ ± 1113	95.0*	

*Byrsonima basiloba* (murici-de-ema)	0 (control)	2.90 × 10^5^ ± 3502	—	42.9
	5	2.92 × 10^5^ ± 8077	0	
	50	0.30 × 10^5^ ± 2376	89.7*	
	100	0.19 × 10^5^ ± 464	93.4*	

*Byrsonima intermedia* (murici-do-campo)	0 (control)	1.43 × 10^5^ ± 9123	—	56.8
	5	1.22 × 10^5^ ± 7767	14.6	
	50	0.57 × 10^5^ ± 3648	60.1*	
	100	0.37 × 10^5^ ± 2373	74.1*	

*Davilla elliptica* (cipó-de-carijó)	0 (control)	2.98 × 10^5^ ± 16869	—	41.1
	5	2.80 × 10^5^ ± 15811	6.0	
	50	0.29 × 10^5^ ± 1651	90.3*	
	100	0.16 × 10^5^ ± 891	94.6*	

*Qualea grandiflora* (pau-da-terra)	0 (control)	2.18 × 10^5^ ± 7691	—	39.3
	5	1.95 × 10^5^ ± 6878	10.5	
	50	0.15 × 10^5^ ± 867	93.1*	
	100	0.14 × 10^5^ ± 765	93.6*	

*Qualea parviflora* (ipê-cascudo)	0 (control)	2.20 × 10^5^ ± 18681	—	27.2
	5	1.14 × 10^5^ ± 9655	48.0*	
	50	0.14 × 10^5^ ± 1194	93.6*	
	100	0.11 × 10^5^ ± 931	95.0*	

*Qualea multiflora* (cerrado-campo)	0 (control)	2.60 × 10^5^ ± 22417	—	27.7
	5	1.37 × 10^5^ ± 11586	47.3*	
	50	0.17 × 10^5^ ± 1432	93.5*	
	100	0.13 × 10^5^ ± 1117	95.0*	

*Mouriri pusa * (puçá)	0 (control)	2.30 × 10^5^ ± 4038	—	42.4
	5	2.30 × 10^5^ ± 4127	0	
	50	0.18 × 10^5^ ± 520	92.2*	
	100	0.16 × 10^5^ ± 459	93.0*	

Quercetin (standard)	0 (control)	2.48 × 10^5^ ± 5987	—	<1.0
	1	1.11 × 10^5^ ± 3211	55.2*	
	5	0.18 × 10^5^ ± 1101	92.7*	
	50	0.15 × 10^5^ ± 578	94.0*	
	100	0.13 × 10^5^ ± 499	94.8*	

^a^Integrated area of chemiluminescence curve: mean of triplicate readings ± SD (*n* = 3); ^b^compared to the control; ^c^efficient concentration; *statistically significant difference (*P* < 0.05).
